# The effect of pre-pregnancy body mass index and gestational weight gain on pregnancy outcomes in urban care settings in Urmia-Iran

**DOI:** 10.1186/1471-2393-6-15

**Published:** 2006-04-20

**Authors:** Zahra Yekta, Haleh Ayatollahi, Reza Porali, Azadeh Farzin

**Affiliations:** 1 Community Medicine Department, Faculty of Medicine, Urmia University of Medical Sciences,, Urmia, Iran; 2 Department of Obstetric and Gynecology, Faculty of Medicine, Urmia University of Medical Sciences, Urmia, Iran; 3 Department of Health Education, Urmia University of Medical Sciences, Urmia, Iran; 4 University of Cincinnati College of Medicine, Cincinnati Children's Hospital Medical Center, Cincinnati, OH,USA

## Abstract

**Background:**

Nutritional status of women has been considered an important prognostic indicator of pregnancy outcomes. Few studies have evaluated patterns of weight gain and pre-pregnancy body mass index in developing regions where malnutrition and poor weight gain as well as maternal obesity have significant influences on the pregnancy outcome. This study aims to show effect of pregnancy body mass index and the corresponding gestational weight gain on the outcome of pregnancy.

**Methods:**

On a prospective cross sectional study, two hundred and seventy women from urban areas of Northwest Iran were recruited for participation during their first eight weeks of pregnancy. Body mass index (BMI) was categorized and gestational weight gain was divided into two groups of normal and abnormal based on recommendations of Institute of Medicine (IOM) published in 1990. Chi square and one way ANOVA were used in the univariate analysis of the association between weight gain and corresponding adverse outcomes including cesarean, preterm labor and low neonatal birth weight. Adjusted odds ratios for adverse outcomes were determined by multiple logistic regression models, while controlling for the following factors: maternal age, parity, and education.

**Results:**

Both pre-pregnancy BMI < 19 and abnormal weight gain during pregnancy were found to be associated with low neonatal birth weight defined as < 2500 g. Abnormal weight gain, during pregnancy was not related to an increased risk of preterm labor or cesarean delivery but it was highly associated with low birth weight (LBW)(P < 0.05).

**Conclusion:**

Low pre-pregnancy BMI is an established risk factor for LBW. Abnormal gestational weight gain may further complicate the pregnancy as an additional risk factor for neonatal LBW. All women, regardless of their pre-pregnancy BMI may be at risk for abnormal weight gain and hence low birth weight. Pre-pregnancy and gestation nutritional assessments remain significant part of all prenatal visits.

## Background

Maternal nutritional status is important for health and quality of life in women and their growing fetus. Maternal pre-pregnancy nutritional status and pregnancy weight gain also affect the health and survival of the newborn. Consequently, various recommendations have been made about weight gain during pregnancy [1]. The Institute of Medicine (IOM) report which released in 1990, categorized according to the pre-pregnant Body Mass Index (BMI) (table 1). This report confirmed a strong association between weight gain during pregnancy and infant size [2]. Since the publication of the initial report, a large body of literature has continued to accrue, which addressing not only birth weight but also other outcomes related to labor, delivery, and maternal postpartum weight status [3].

**Table 1:** Recommended ranges of total weight gain for pregnant women by pre-pregnancy body mass index (from the Institute of Medicine 1990)

**BMI Level**	**Recommended weight gain**
< 19.8	12.5–18
19.8–26	11.5–16
26–29	7–11.5
> 29	> 7

The relationship between maternal obesity and adverse pregnancy outcome has been well characterized in obstetric and public health literature [4]. Women with lower than normal maternal body weight have also been shown to be at increased risk for adverse prenatal outcomes such as prematurity and intrauterine growth restriction [5].

Low birth weight (LBW) defined as birth weight less than 2500 grams, and is an important determinant of infant mortality and morbidity [6]. A strong relationship between maternal pregnancy weight gain and birth weight has been demonstrated consistently, and low maternal weight gain is considered as a preventable risk factor for LBW [6].

However, weight gain in most pregnant women is not within the range recommended by IOM, and is considered to be too low or too high compared with current standards [3]. Furthermore, information on patterns of weight gain in pregnant women from developing countries is scarce [1].

The purpose of our study is to describe the patterns of gestational weight gain and to demonstrate their effects on both maternal and neonatal outcomes in urban care settings. This understanding may lead to more consistent and evidence-based recommendations for desirable prepregnancy and gestational nutritional status to the expecting mother.

## Methods

A prospective cross sectional study was initiated in 2002. Pregnant women who enrolled in public health care centers in urban areas of Urmia were selected for a longitudinal study. Initially we selected eight health care centers among the total of 18 centers as cluster sampling, and then 34 subjects meeting our criteria were selected and recruited from each health care center.

Between the years 2002 and 2003, a cohort of 270 women in their first eight weeks of pregnancy were recruited to participate in the study. Although enrolled subjects did not receive any additional interventions during their pregnancy, and received the standard prenatal care as usual based on health care center rules, ethics committee approval was obtained from health deputy, everyone had the right to participate in our survey and to be freedom to leave study, whenever they wanted. Women were informed about the study. All participants and their husband were full consent of entering our study. Information was kept secret. In all subjects the date of last menstrual period (LMP) was recorded and suspected pregnancies were confirmed with a pregnancy test. Estimated gestational age was calculated based on the recalled LMP and ultrasound studies. Baseline weight and height were recorded during the initial visit. Trained field workers visited the women at the health care centers at least once a month during their pregnancy to conduct interviews and obtain gestation weight gain. Body mass was measured with a calibrated scale accurate to within 0.5 kg while subjects were wearing the possible lightest clothing. Pre-pregnancy weight was based on the weight measured during at least the first two months of pregnancy, and confirmed with maternal recall at the fist visit. Several studies have reported that recalled pre-pregnancy weight reflects actual weight in women [7,8]. Subjects with complicated pregnancies such as pre-eclampsia, twin gestation, history of diabetes, cardiovascular and kidney diseases were excluded and replaced by new subjects.

Information on maternal age, parity and education was collected. Maternal pre-pregnancy body mass index were categorized based on the 1990 IOM standards of desirable weights (ref 2, table 1). Total pregnancy weight gain was estimated by subtracting the pre pregnancy weight from the last measured weight before delivery. Weight gain in relation to pre-pregnancy BMI was divided into two groups of normal and abnormal based on recommendation of IOM. Accordingly normal group is defined as a weight gain within the suggested range and abnormal one as above or below the recommendation. The influence of gestational weight gain on maternal and neonatal outcomes including preterm delivery (gestational age < 37 weeks), LBW, and cesarean delivery were evaluated.

Chi square and one way ANOVA were performed as appropriate in two – tailed analyses. P- value less than 0.05 was considered as being significant. Logistic regression analysis was also used to examine the relationship between weight gain and outcomes of pregnancy. Weight gain was modeled as numerical variable with normal and abnormal weight gain. All variables including weight gain, education, age group, parity and prepregnancy BMI were entered as potentially confounding variables and, then to obtain odds ratios, adjusted for the significant predictors of adverse outcomes listed as preterm delivery, low birth weight and cesarean delivery.

## Results

A total of 270 women participated in the study with a mean age of 32.3 ± 4.9 years. Of those, 85.2% (230 cases) were between ages 19–35 yr, 7% were under than 18 yr, and 7.8% were over 35 years of age. The majority (79.1%) of subjects were multiparous, and the remaining (21.9%) nulliparous. Further demographic information is listed on table 2.

**Table 2:** Characteristics of pregnant women and adverse outcome frequency

	**Frequency (%)**	**Mean**
**BMI**		
< 19.8	30 (11.1)	**-**
19.8–26	140 (51.9%)	
26.1–29	52 (19.3%)	
> 29	48 (17.8%)	
**Age group**		
< 18	19 (7%)	26.5 ± 6
19–34	230 (85.2%)	
> 34	21 (7.8%)	
**Parity**		
Nuliparous	60 (21.9%)	-
Muliparous	210 (79.1%)	
**Education**		
Illiterate	65 (24.1%)	-
High School	164 (60.7%)	-
University	41 (15.2%)	-
**Normal weight gain**	114 (42.2%)	-
		
**Weight gain**	-	8.8 ± 4.1
**Birth weight**	-	3276.4 ± 546
		
**Low birth weight**	26 (9.6%)	
**Preterm labor**	16 (5.9%)	-
**Cesarean section**	77 (28.5%)	-

Pre-pregnancy BMI were categorized based on IOM recommendations. The mean neonatal birth weight was 3483 ± 425 grams with 90.4% of newborns with a birth weight over = 2500 grams as listed in table 2. Neonatal birth weight varied significantly based on maternal prepregnancy BMI. Women with BMI < 19.8 kg/m^2 ^delivered neonates with average birth weight of 3102 ± 487 grams in comparison with the average neonatal birth weight of 3469 ± 588 grams among neonates of women with BMI > 30 kg/m^2^. Therefore 16.7% of all neonates of women with BMI < 19.8 were LBW, but only 4% of newborns of women with a BMI > 30 kg/m^2 ^were categorized as LBW (p < 0.05) (Table 3).

**Table 3:** Adverse outcome based on pre-pregnancy BMI

**Pre-pregnancy BMI\Adverse outcome**	**Neonate Weight**		**Preterm**	**Cesarean**
			
	**LBW**	**Mean**		
< 19.8	5 (16.7%)	3102.6	2(6.7%)	4(13.3%)
19.8–26	15(10.7%)	3256.4	8(5.7%)	38(27.1%)
26–29	4(7.7%)	3252.1	4(7.7%)	17(32.7%0
> 29	2(4.2%)	3469.7	2(4.2%)	18(37.5%)

A total of 28.5% of women required cesarean delivery. Women with BMI < 19.8 kg/m^2 ^had the lowest rate of cesarean section (13.3%), and the most cesarean interventions were performed among subjects categorized as a group with pre-pregnancy BMI > 30 kg/m^2^. The overall incidence of preterm delivery was 5.9% (Table 3).

**Figure 1:**
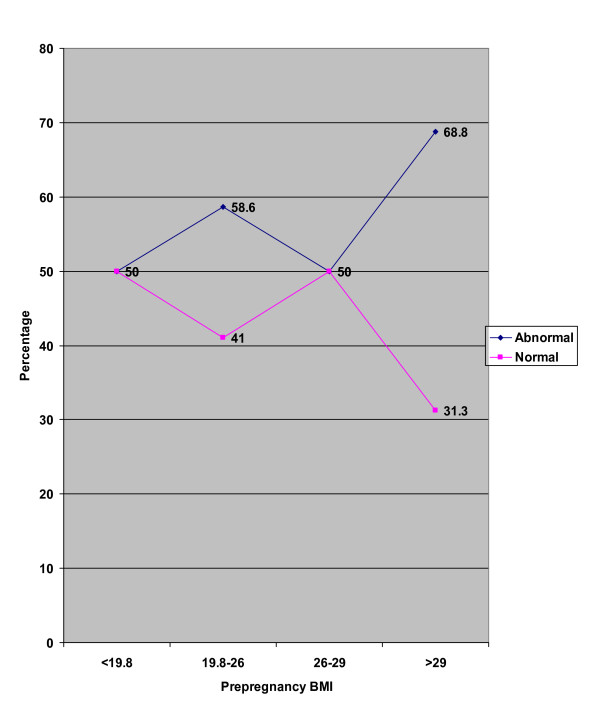
Relation between pre-pregnancy BMI and weight gain.

**Figure 2:**
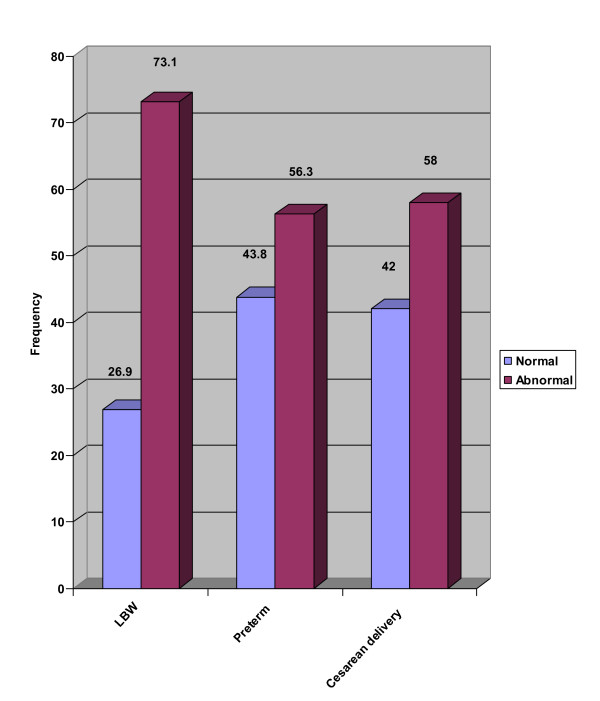
Incidence of LBW, Preterm delivery and cesarean delivery with normal and abnormal pregnancy weight gains (recommendations of the Institute of Medicine).

**Figure 3:**
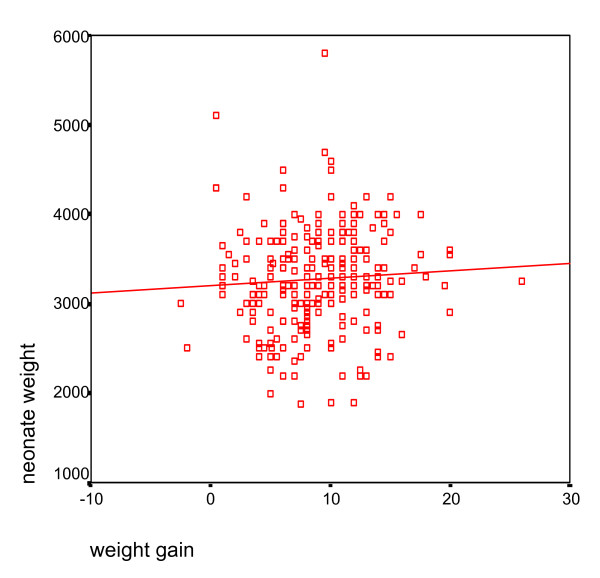
Correlation between weight gain during pregnancy and neonate weight.

The mean weight gain in the four prepregnancy BMI groups (< 19.8, 19.8–26, 26–29, > 30 kg/m^2^) were respectively 9.7 ± 3.5, 9.3 ± 4.3, 7.7 ± 3.5, and 11.2 ± 4.1 kg as listed in table 4. One hundred and fifteen (42.6%) subjects reached IOM recommended weight gain (Figure 1). The incidence of LBW, preterm delivery and cesarean delivery with normal and abnormal pregnancy weight gains are listed in figure 2. Correlation between weight gain and neonate weight are shown in figure 3. Maternal age was not considered as a significant variable in extent of weight gain with 26% of subjects with normal weight gain belonged to the high risk age group (< 18 years old or > 35 years old), and remaining 74% were between ages 18 and 35 yr. We were not able to demonstrate abnormal gestational weight gain as a risk factor for preterm labor (6.1 versus 5.8) or cesarean delivery (30.7) versus (26.9%) with both p values more than 0.05. Abnormal maternal weight gain during pregnancy was highly associated with LBW (12.2 vs 6.1% with p < 0.05). We furthermore utilized multiple logistic regression analysis to estimate the association between abnormal weight gain during pregnancy and the risk of adverse outcomes while controlling for the effect of potentially confounding variables. A clear and significant relationship was seen between abnormal weight gain and LBW with OR: 2.37 CI: (1.7–3.2) (p < 0.05). Therefore, this study suggests a steady decrease in the incidence of LBW as mean pregnancy weight gain increases. Low level of education and prepregnancy BMI under 19.8 kg/m^2 ^were associated significantly with cesarean delivery as an adverse outcome with an OR: 1.39 and 1.72 respectively (p < 0.05).

**Table 4:** Maternal weight gain based on pre-pregnancy BMI

**Pre-pregnancy BMI\Weight gain**	**Normal weight gain **(within the IOM ranges)	**Abnormal weight gain**		**Mean**
			
		**Low weight gain**	**High weight gain**	
< 19.8	15(50%)	15(50%)	0(0%)	9.7 ± 3.5
19.8–26	58(41.4%)	77(55%)	5(3.6%)	9.3 ± 4.3
26–29	26(50%)	23(44.2%)	3(5.8%)	7.7 ± 3.5
> 29	16(33.3%)	9(18.8%)	23(47.9%)	11.2 ± 4.1
				

Total	115(42.6%)	124(45.9%)	31(45.9%)	8.8 ± 4.1

## Discussion

Maternal BMI and gestational weight gain reflect nutritional status before and during pregnancy. Some evidence has considered abnormal weight gain to have a significant relationship with poor pregnancy outcomes. However, weight gain in most pregnant women is not within normal range suggested by IOM [3].

For instance, our data indicates only half the subjects had a normal prepregnancy BMI (19.8–26 kg/m^2^). Furthermore, 50% of women with a normal BMI did not ultimately achieve the recommended weight gain. Abnormal weight gain was also seen in more than half of obese women (BMI > 29 kg/m^2^). Overall, there was no significant difference between the initial BMI and percentage of desired gestational weight gain.

Although 60% of low educated subjects were recognized with abnormal weight gain, we could not find any significant difference between weight gain and educational level. Although level of education did not influence weight gain significantly, but illiterate subjects were at higher risk for poor weight gain. This is perhaps explained by patient compliance and access to nutritional counseling and resources.

Previous studies have shown that pregnancy weight gain within the ranges recommended by IOM is associated with the best outcome for both mothers and infants [9,10]. On the contrary, some studies, that retrospectively assessed the sensitivity and specificity of this indictor, concluded that maternal weight gain alone is neither a sensitive nor a specific predictor of poor pregnancy outcomes [3]. Rasmussen et. al reported that constitutional low weight for height is not a predictor of complications during delivery, and no special observation of this group is recommended [11].

Since the amount of total weight gain is widely variable among women with good pregnancy outcomes, and the perinatal outcomes of interest are multifactorial in origin, it should not be expected for weight gain alone to be utilized as a perfect diagnostic or screening tool [4]. Our study suggests that deviation in maternal weight gain can act as a useful marker of newborn weight at birth and, also pre-pregnancy BMI can predict fetal weight especially in women with BMI < 19.8 kg/m^2^. Ogunyemi et. al has mentioned that normal BMI and ideal weight gain in pregnancy is associated with decreased perinatal complications and an optimum birth weight [9]. Another study showed that being moderately underweight was not associated with increased risk of adverse pregnancy outcomes, but being severely underweight was an important risk factor for reduced fetal growth [12].

Many studies looked more closely at the association between pregnancy weight gain and the rates of cesarean delivery.

In our study, weight gain was not associated with increased cesarean section rates, but frequency of cesarean section was significantly different among women in different levels of pre-pregnancy BMI, where, obese women experiencing the highest rate of cesarean. This is most likely explained based on the high incidence of large infants in this group. Another study showed that overweight status (25.0= BMI < 30.0 kg/m^2^) and obesity (BMI = 30.0 kg/m^2^) are only weak predictors of labor complications [13].

Steinfeld et.al reported obese Hispanic and African American women were more likely than obese white women to deliver by cesarean (P = 0.03). Therefore racial differences affect the complication rates in obese women, and may also influence prenatal counseling and pregnancy management [14]. It is important to consider the underlying issues in controversy, as maternal anthropometry differs across ethnic groups and therefore different recommendations should be made for specific populations.

The relation between low pregnancy weight gain and increased risk of preterm birth was previously illustrated by Caminchael et. al [15]. Although the biological mechanism underlining this association is unknown, it appears that a rate of pregnancy weight gain below the lower limit of the IOM's recommended range especially in late pregnancy may be related to a higher risk of preterm birth [3]. Other studies have found that risk of preterm birth was not associated with maternal BMI [12]. In our study there was no difference between weight gain and preterm delivery. This may be due to our data collection as we did not analyze weight fluctuations weekly, and therefore we were not able to assess any acute inappropriate weight gains, which may have occurred during period of a week.

## Conclusion

Our data support the commonly recommended notion that health care workers and pregnant women should continue to follow patterns of weight gain during pregnancy regardless of pre-pregnancy BMI. Efforts should be directed to attain adequate prepregnancy weight and maintain recommended weight gain to reduce the likelihood of LBW babies. Nutritional education may be effective on improving weight gain during pregnancy. Special attention should be paid to women with low prepregnancy BMI and abnormal weight gain as well as illiterate women who are at higher risk for poor weight gain.

## Competing interests

The author(s) declare that they have no competing interest

## Authors' contributions

**ZY **conceived the study and participated in its design and performed the statistical analysis.

**HA **participated in the designing of the study and training health care workers.

**RP **supervised health care workers and coordinated them.

**AF **helped to draft the manuscript.

All authors read and approved the final manuscript.

## Pre-publication history

The pre-publication history for this paper can be accessed here:


